# The association between trajectory of change in social functioning and psychological treatment outcome in university students: a growth mixture model analysis

**DOI:** 10.1192/j.eurpsy.2024.1475

**Published:** 2024-08-27

**Authors:** P. Barnett, R. Saunders, J. E. Buckman, S. A. Naqvi, S. Singh, J. Stott, J. Wheatley, S. Pilling

**Affiliations:** ^1^Psychology and Language Sciences, UCL; ^2^National Collaborating Centre for Mental Health, Royal College of Psychiatrists; ^3^ Camden and Islington Foundation trust; ^4^ North East London NHS Foundation Trust; ^5^Homerton University Hospital NHS Foundation Trust, London, United Kingdom

## Abstract

**Introduction:**

Attendance at university can result in social support network disruption. This can have a negative impact on the mental health of young people. Demand for mental health support continues to increase in universities, making identification of factors associated with poorer outcomes a priority. Although social functioning has a bi-directional relationship with mental health, its association with effectiveness of psychological treatments has yet to be explored.

**Objectives:**

To explore whether students showing different trajectories of change in social function over the course of treatment differed in eventual treatment outcome.

**Methods:**

Growth mixture models were estimated on a sample of 5221 students treated in routine mental health services. Different trajectories of change in self-rated impairment in social leisure activities and close relationships (Work and Social Adjustment Scale (WSAS) items 3 and 5) during the course of treatment were identified. Associations between trajectory classes and treatment outcomes were explored through multinomial regression.

**Results:**

Five trajectory classes were identified for social leisure activity impairment (Figure 1), and three classes were identified for close relationship impairment (Figure 2). For both measures the majority of students remained mildly impaired (Class 1). Other trajectories included severe impairment with limited improvement (Class 2), severe impairment with delayed improvement (Class 3), and, in social leisure activities only, rapid improvement (Class 4), and deterioration (Class 5). There was an association between trajectories of improvement in social functioning over time and positive treatment outcomes. Trajectories of worsening or stable severe impairment were associated with negative treatment outcomes.

**Image:**

**Figure:**
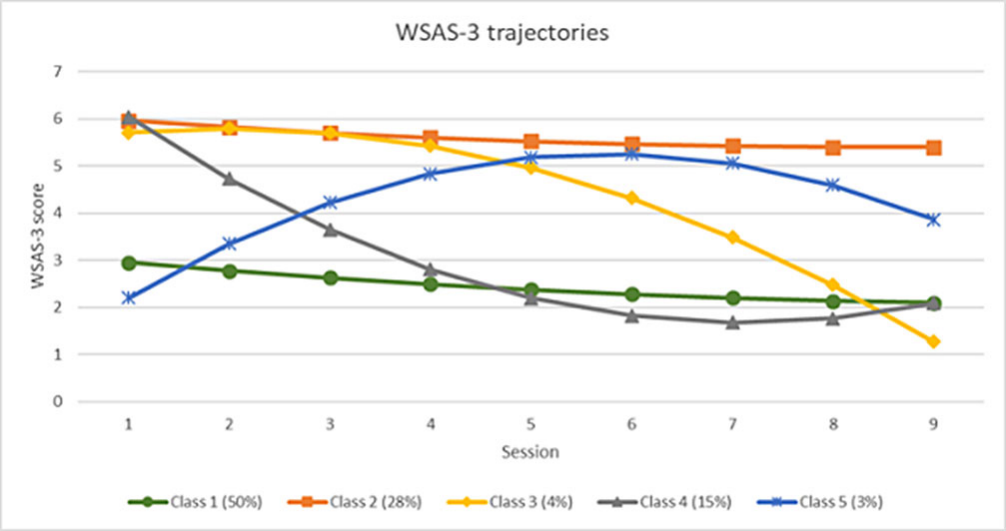

**Image 2:**

**Figure:**
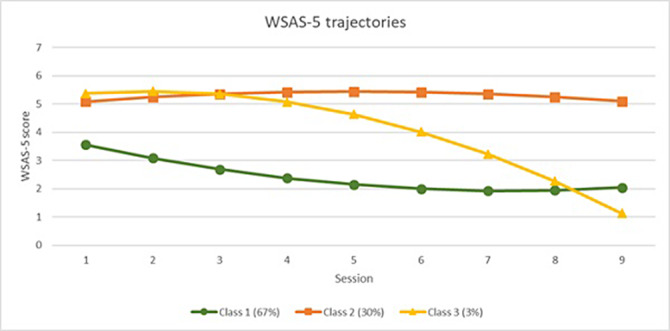

**Conclusions:**

Changes in social functioning impairment are associated with psychological treatment outcomes in students, suggesting that these changes may be associated with treatment effectiveness or recovery experiences. Future research should look to establish whether a causal link exists to understand if additional benefit for students can be gained through integrating social support within psychological treatment.

**Disclosure of Interest:**

None Declared

